# Hypomagnesaemia with varying degrees of extrarenal symptoms as a consequence of heterozygous *CNNM2* variants

**DOI:** 10.1038/s41598-024-57061-7

**Published:** 2024-03-22

**Authors:** Willem Bosman, Gijs A. C. Franken, Javier de las Heras, Leire Madariaga, Tahsin Stefan Barakat, Rianne Oostenbrink, Marjon van Slegtenhorst, Ana Perdomo-Ramírez, Félix Claverie-Martín, Albertien M. van Eerde, Rosa Vargas-Poussou, Laurence Derain Dubourg, Irene González-Recio, Luis Alfonso Martínez-Cruz, Jeroen H. F. de Baaij, Joost G. J. Hoenderop

**Affiliations:** 1 Department of Medical BioSciences, Radboudumc, Nijmegen, The Netherlands; 2 Division of Pediatric Metabolism, Cruces University Hospital, CIBER-ER, Metab-ERN, University of the Basque Country (UPV/EHU), Biobizkaia Health Research Institute, Barakaldo, Spain; 3 Pediatric Nephrology Department, Cruces University Hospital, CIBERDEM, CIBER-ER, Endo-ERN, Biocruces Bizkaia Health Research Institute and University of the Basque Country (UPV/EHU), Barakaldo, Spain; 4 Deparment of Clinical Genetics, Erasmus MC, Rotterdam, The Netherlands; 5 Discovery Unit, Department of Clinical Genetics, Erasmus MC, Rotterdam, The Netherlands; 6 ENCORE Expertise Center for Neurodevelopmental Disorders, Erasmus MC, Rotterdam, The Netherlands; 7 Department of General Pediatrics, Erasmus Medical Center Sophia Children’s Hospital, Rotterdam, The Netherlands; 8 Unidad de Investigación, Renal Tube Group, Hospital Universitario Nuestra Señora de Candelaria, Santa Cruz de Tenerife, Spain; 9 Department of Genetics, University Medical Center Utrecht, Utrecht, The Netherlands; 10 Service de medecine genomique des maladies rares, AP-HP, universite Paris Cité, Paris, France; 11 Centre de reference des maladies renales hereditaires de l’enfant et de l’adulte MARHEA, hopital Européen Georges Pompidou, Paris, France; 12 CNRS, centre de recherche des Cordeliers, Inserm UMRS 1138, Sorbonne universite, universite Paris Cité, Paris, France; 13 Hôpital Édouard Herriot, Hospices civils de Lyon, service de nephrologie, dialyse, hypertension et exploration fonctionnelle renale, Lyon, France; 14 Centre de reference des maladies renales rares et phosphocalciques, Nephrogones, Hôpital Femme-Mère-Enfant Bron, Bron, France; 15 Faculté de medecine Lyon est, Université Claude Bernard Lyon 1, Villeurbanne, France; 16 Center for Cooperative Research in Biosciences (CIC bioGUNE), Bizkaia Science and Technology Park, Derio, Bizkaia Spain

**Keywords:** CNNM2, Variant characterisation, Genetic hypomagnesaemia, Intellectual disability, Genetic testing, Nephrons, Structural biology

## Abstract

Variants in the *CNNM2* gene are causative for hypomagnesaemia, seizures and intellectual disability, although the phenotypes can be variable. This study aims to understand the genotype–phenotype relationship in affected individuals with *CNNM2* variants by phenotypic, functional and structural analysis of new as well as previously reported variants. This results in the identification of seven variants that significantly affect CNNM2-mediated Mg^2+^ transport. Pathogenicity of these variants is further supported by structural modelling, which predicts CNNM2 structure to be affected by all of them. Strikingly, seizures and intellectual disability are absent in 4 out of 7 cases, indicating these phenotypes are caused either by specific *CNNM2* variant only or by additional risk factors. Moreover, in line with sporadic observations from previous reports, *CNNM2* variants might be associated with disturbances in parathyroid hormone and Ca^2+^ homeostasis.

## Introduction

Pathogenic variants in the Cyclin and CBS domain divalent metal cation transport mediator 2 (*CNNM2*) gene are causative for hypomagnesaemia, seizures and impaired intellectual development (MIM #613882/MIM #616418). To date, 13 reports of 26 *CNNM2* variants in individuals with serum magnesium (Mg^2+^) levels of 0.33–0.74 mmol/L (normal 0.7–1.05 mmol/L) have been reported^1–13^. The hypomagnesaemia is of renal origin, as excretion of Mg^2+^ is expected to decrease upon low serum Mg^2+^, but was found to be either normal or increased^1–3,5,11,12^. Epilepsy and intellectual disability (ID) are observed in most, but not all cases. Additional features of *CNNM2*-related disorders seen in subgroups of affected individuals include obesity and motor skill defects. It occurs most frequently as an autosomal dominant disorder, though three cases with homozygous *CNNM2* variants have been described^2,3^.

CNNM2 is an important player in systemic Mg^2+^ homeostasis. It is part of the CNNM family of membrane proteins that share a homologous domain with the prokaryotic Mg^2+^ transporter CorC^14^. The different CNNM proteins show specific expression patterns. CNNM2 is prominently expressed in the brain and in the distal convoluted tubule (DCT) of the kidney^14,15^. By reabsorbing Mg^2+^, sodium and chloride, the DCT plays a key role in regulating electrolyte levels and blood pressure. CNNM2 function is in line with its expression in the DCT and brain, as heterozygous knockout (*Cnnm2*^+/−^) mice develop hypomagnesaemia due to decreased Mg^2+^ reabsorption, while a full knockout (*Cnnm2*^−/−^) is perinatally lethal and leads to brain malformations in a subgroup of mice^16^.

The age-of-onset, phenotypes and symptom severity of individuals with pathogenic *CNNM2* variants can vary substantially, complicating diagnosis and management of this disease. The phenotypes of affected individuals with heterozygous variants range from isolated hypomagnesaemia without clinical symptoms to severe hypomagnesaemia with seizures, ID, motor skill defects and obesity. Furthermore, many reported *CNNM2* variants lack functional studies to confirm pathogenicity. The aim of this study is to improve diagnosis and understanding of genotype–phenotype relationships for *CNNM2*-related disorders by characterising pathogenic variants. Five new variants, as well as two variants from a previous study^4^, were functionally and structurally assessed to determine pathogenicity.

## Materials and methods

### Study participants

Individuals were included when a rare, uncharacterised variant in *CNNM2* was identified during genetic diagnostics that was suspected to be causative for their phenotypes (hypomagnesaemia and/or ID). In addition, we included four uncharacterised variants (NP_060119.3:p.S186T, p.I260F, p.D335N, p.S795*) from a previous study that lacked functional confirmation of pathogenicity^4^. p.S186T and p.I260F as well as novel variants p.E184G and p.T331I did not affect CNNM2 Mg^2+^ transport function (Fig. S1) and were not included for the phenotyping, resulting in seven cases.

### Ethics declarations

Recruitment of participants was part of routine diagnostics performed at the various medical centers in accordance with the guidelines and regulations of the respective center. No additional procedures were required for inclusion in this study. In accordance with the Declaration of Helsinki, written informed consent for the genetic analysis was obtained from all recruited individuals and/or their legal guardians. In addition, oral consent was obtained for the use of anonymised clinical data.

### Genetic analysis

The genetic diagnosis was performed using whole exome sequencing (WES) and a gene panel of known causative genes for hypomagnesaemia (cases 3–5; Table 1 from^17^), ID (case 1 and 2; in-house panels of 1226 and 1235 genes), and/or nephrolithiasis (case 4; in-house panel) at the respective referring institutes: Department of Clinical Genetics, Erasmus MC, Rotterdam, the Netherlands as described previously^18^ (case 1); Department of Genetics, UMC Utrecht, Utrecht, the Netherlands using the SureSelect Crev2 (Agilent) target enrichment set (case 2); Unidad de Investigación, Hospital Universitario Nuestra Señora de Candelaria, Santa Cruz de Tenerife, Spain in collaboration with Macrogen Inc. (Seoul, South Korea) using a SureSelect V6 Library and the NovaSeq 6000 system (Illumina) for sequencing (case 3); Department of Genetics, Radboudumc, Nijmegen, the Netherlands in collaboration with BGI-Europe (Copenhagen, Denmark) using the SureSelectXT Human All Exon v5 kit (Agilent) for exome capture and the HiSeq2000TM (Illumina) for sequencing (case 4); Molecular Genetic Laboratory, Biocruces Bizkaia Health Research Institute, Barakaldo, Spain using the DNA Prep with Enrichment kit (Illumina) and the NovaSeq 6000 sequencer (case 5). In-house pipelines were used for variant calling, annotation and prioritization at the respective institutes. In general, reads were aligned to human reference genome GRCh37/hg19 using Burrows-Wheeler Alignment^19^ and variants were called using the Genotype Analysis Toolkit HaplotypeCaller^20^. The following filtering strategy was applied: ≥ 5 reads, variant in ≥ 20% of reads, minor allele frequency < 0.1%, coding region or canonical splice site only, only non-synonymous (including nonsense) variants or insertions and deletions (including frameshift variants). Allele frequencies were assessed in gnomAD v2.1.1, Exome Variant Server v.0.0.30 and GME Variome^21–23^ in May 2023. To predict deleteriousness in silico, MutationTaster2021^24^ was used as well as PhyloP^25^, CADD v1.6^26^, PolyPhen-2^27^ and VEST-4^28^ scores using cut-off values of ≥ 7.367, ≥ 25.3, ≥ 0.978 and ≥ 0.764 respectively, based on the analysis by Pejavar et al*.*^29^. Using the combined results of the genetic, phenotypic, in silico and functional analyses, variants were classified as either benign, likely benign, uncertain significance, likely pathogenic or pathogenic according to the guidelines of the American College of Medical Genetics and Genomics (ACMG)^30–32^.

**Table 1:** Characterisation of the main *CNNM2*-related phenotypes in new cases.

Variant	Case 1	Case 2	Family 3		Family 4		Case 5	Case 6	Case 7
	p.A92P	p.V324L	p.M383V		p.E431K				
			Case 3.1 (index)	Case 3.2 (mother)	Case 4.1 (index)	Case 4.2 (daughter)	p.G437E	p.S795*	p.D335N
Age at onset (years)	After birth	2	5	NA	50	21	4	After birth	16
Hypo-magnesemia (mmol/L)	N (0.73)	Y (0.55–0.57)	Y (0.62)	Y (0.6)	Y (0.52)	Y (0.5)	Y (0.53–0.62)	Y (0.57)	N (0.7)
Seizures	N	Y	N	N	N	N	N	N	N
Intellectual disability	Y	Y	Y	N	N	N	N	N	N
Obesity	Y	Y	N	N	N	N	N	N	N
Motor skill defects	Y	N	N	N	N	N	N	N	N
Hyper-parathyroidism	N	N	N	N	Y	N	NA	Y	Y
Other		ASD	ADHD, dyslexia		Hypercalcaemia		ADHD, dyslexia, multiple thyroid colloid cysts	Hypocalciuric hypercalcemia	Hypocalciuria
Comments					*SLC9A3R1* variant				VUS in *VDR*

ADHD, attention deficit hyperactivity disorder; ASD, autism spectrum disorder; N, no; SLC9A3R1, Solute carrier family 9 member 3 regulator 1; VDR, vitamin D receptor; VUS, variant of unknown significance; Y, yes.

### DNA constructs

The human *CNNM2* coding sequence (NM_017649.5) was cloned into the pCIneo-IRES-mCherry backbone with a carboxyl-terminal FLAG-tag. To generate the missense variants, the Q5 site-directed mutagenesis kit (New England BioLabs; E0554) was used according to manufacturer’s instructions. For the p.Ser795* variant, the truncated coding sequence was cloned into the same backbone, placing the FLAG-tag before the new stop codon.

### Cell culture

HEK293 cells (a kind gift from the Laboratory of Ion Channel Research, KU Leuven) were cultured in DMEM (Gibco, 42430-035) supplemented with 10% (v/v) FBS, 1 mM sodium pyruvate and 0.1 mM non-essential amino acids (Westburg, CA NEAA-B) at 37 °C, 5% (v/v) CO_2_. For transfection, Lipofectamine 2000 (Invitrogen, 11668-019) was used at a 1:2 DNA to Lipofectamine ratio, following manufacturer’s instructions.

### ^25^Mg^2+^ uptake assay

The uptake assay was performed as described previously^2^, using a timepoint of 10 min. ^25^Mg^2+^ uptake levels of cells transfected with empty vector were used for background subtraction. Cells of each condition were lysed for SDS-PAGE and Western blot analysis. Immunoblots were incubated with primary antibodies targeting FLAG (Sigma-Aldrich; F1804) diluted 1:5000 or β-actin (Sigma-Aldrich; A5441) diluted 1:10,000 in 1% (w/v) milk. Peroxidase-conjugated secondary antibody (Jackson ImmunoResearch; 515-035-003) diluted 1:10,000 and PICO chemiluminescent substrate (ThermoFisher; 34580) were used for visualization with the ImageQuant LAS 4000 (GE Healthcare). Image processing was performed in ImageJ^33^ and was only used for adjusting brightness and contrast.

### Structural modelling

The analysis of the effects of each variant was conducted by integrating the existing crystallographic knowledge on CNNM family members^34–39^ with the Deep Learning Structural determination software by AlphaFold 2.3.0^40^ using an adapted version of the AlphaFold code (https://github.com/deepmind/alphafold)^41^. DynaMut2 was utilised to analyse the surrounding environment of the respective mutations^42^. Considering the flexibility of the inter-domain connections, our structural calculations were performed on the monomeric and the dimeric associations formed by each of the independent domains, and not in the full-length template. These dimeric associations have been consistently observed in crystals of all CNNMs, reviewed in detail previously^37,39,43^. The graphical representation of the proteins’ three-dimensional structures was performed using PyMOL^44^.

### Statistics

Statistical significance was tested using unpaired t-tests with Welch’s correction. Based on the Bonferroni method, a *p-*value < 0.0071 was considered statistically significant to correct for multiple testing.

## Results

### Characteristics of affected individuals

The phenotypes of seven individuals or families with heterozygous *CNNM2* variants was examined, particularly focusing on the most common phenotypes associated with CNNM2-related disorders (Table 1). Each case is discussed below.

Case 1 is an 8-year-old girl with attention deficit hyperactivity disorder (ADHD), obesity (BMI > 95th percentile) and developmental delay in speech and gross motor skills. Mild ID is suspected on clinical grounds and school performances, with a recently measured total IQ of 76 (verbal 98, working memory 67, processing speed 72), although earlier IQ assessments were higher (verbal 91, performance 87, total 85). A single nucleotide polymorphism-array and routine metabolic investigations in serum and urine were unremarkable. Serum electrolyte levels are normal, although her Mg^2+^ levels are towards the low end of the normal range (0.71–0.73 mmol/L). She does not suffer from seizures. WES trio analysis, with a focused analysis of a virtual ID gene panel revealed a de novo variant NM_017649.5:c.274G>C (NP_060119.3:p.A92P) in *CNNM2*, with no other likely disease-explaining candidates.

Case 2 is a 5-year-old boy with obesity, autism and developmental delay in communication skills, as he is not able to speak and only understands simple directions, while motor skills are normal. In addition, he has suffered seizures twice since the age of 2. Blood analysis at the age of 4 showed hypomagnesaemia (0.55 mmol/L) but normal Ca^2+^ and PTH levels. Mg^2+^ supplementation was started, but serum levels remained low at 0.57 mmol/L. While a single nucleotide polymorphism-array was unremarkable, a WES trio analysis revealed the de novo c.970G>C (p.V324L) variant in *CNNM2*.

Case 3 presented at the clinic at the age of 5 with ADHD, mild ID and developmental language disorder. Further analysis revealed the presence of hypomagnesaemia (0.62 mmol/L). A variant in *CNNM2* was identified after WES trio analysis (c.1147A>G, p.M383V). This variant was inherited from the mother, who also has hypomagnesaemia (0.60 mmol/L) but none of the other symptoms. Seizures, motor skill defects and obesity are absent in both.

Case 4 was first evaluated in adulthood because of nephrolithiasis, chondrocalcinosis and hyperparathyroidism, accompanied by hyperplasia of the parathyroid gland. Further analyses revealed hypercalcaemia, hypercalciuria, hypophosphataemia, osteopenia and hypomagnesaemia (0.52 mmol/L). Subsequently, her daughter was also screened and was found to have hypomagnesaemia (0.5 mmol/L), but normal PTH and phosphate levels. The daughter also is overweight (BMI 85–95th percentile). A variant in *CNNM2* (c.1291G>A, p.E431K) was found in both after analyzing WES data using hypomagnesaemia and nephrolithiasis gene panels. Targeted Sanger sequencing of CNNM2 showed that this variant is absent in the mother of the index case, who has normal Mg^2+^ levels (0.75 mmol/L). In addition, a known variant in *SLC9A3R1* (NM_004252.5:c.328C>G, p.L110V) was identified in the index case, but not the daughter. This variant and other variants in this gene have been linked to hypophosphataemia with nephrolithiasis or osteoporosis^45,46^.

Case 5 presents with hypomagnesaemia (0.53–0.62 mmol/L) along with dyslexia and ADHD. He also has multiple thyroid colloid cysts, but normal thyroid function. ID, seizures, obesity and motor skill defects are not present. Genetic screening revealed a variant c.1310G>A (p.G437E) in *CNNM2* when he was 9 years old. Targeted Sanger sequencing of *CNNM2* in the parents confirmed a de novo origin.

Cases 6 and 7 have been described previously^4^. In short, case 6 has hypocalciuric hypercalcaemia, hyperparathyroidism and hypomagnesaemia (0.57 mmol/L). The *CNNM2* variant is c.2384C>A (p.S795*). ID or motor skill defects are not reported, although neuropsychological tests have not been performed. Case 7 also presented with hypocalciuria and hyperparathyroidism, as well as low 25-hydroxyvitamin D levels, nephrocalcinosis and low-normal Mg^2+^ levels (0.7 mmol/L). Gene panel analysis revealed the de novo c.1003G>A (p.D335N) variant in *CNNM2* and a heterozygous variant in the vitamin D receptor gene (*VDR;* NM_000376.3:c.889G>A, p.V297I). This *VDR* variant is also present in both parents, though only the father presents with hypocalciuria.

### Functional characterisation

**Figure 1:**
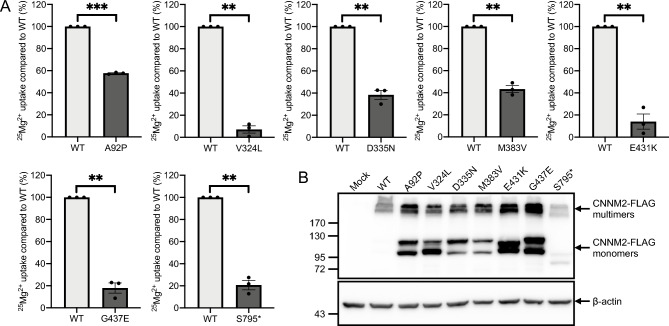
Reduced Mg^2+^ uptake of variants compared to WT CNNM2. (**A**) Percentage of residual ^25^Mg^2+^ uptake capacity of each variant compared to WT. Data were obtained from 3 independent experiments and presented as mean ± SEM. **P < 0.01, ***P < 0.0001. (**B**) Representative Western blot of WT and variant CNNM2 expression.

None of the seven variants are present in the GnomAD, Exome Variant Server or GME Variome databases, while six out of seven are predicted to be deleterious by at least three out of the five in silico tools (Table S1). p.A92P is predicted to be benign by MutationTaster2021 and has scores below the cut-off values for the other prediction tools. To obtain more direct evidence of pathogenicity, we performed an uptake assay with the Mg^2+^ isotope ^25^Mg^2+^. Cells transfected with p.V324L (7.1% of WT, P = 0.001), p.E431K (14.1%, P = 0.006), p.G437E (17.9%, P = 0.003) and p.S795* (20.7%, P = 0.003) all showed a substantial loss of function compared to cells with WT CNNM2 (Fig. 1A). p.A92P (57.7%, P = 0.0001), p.D335N (38.3%, P = 0.004) and p.M383V (43.3%, P = 0.003) showed a less pronounced, but significant reduction in ^25^Mg^2+^ uptake. For the missense variants, the reduction in ^25^Mg^2+^ uptake was not due to decreased expression, as variant CNNM2 even showed higher protein expression than WT (Fig. 1B). The truncated protein resulting from the p.S795* variant did show lower expression, indicating it may be a target for degradation. Notably, WT CNNM2 is almost exclusively present in functional multimers, while all variants result in a relatively high presence of monomers.

### Structural modelling

**Figure 2:**
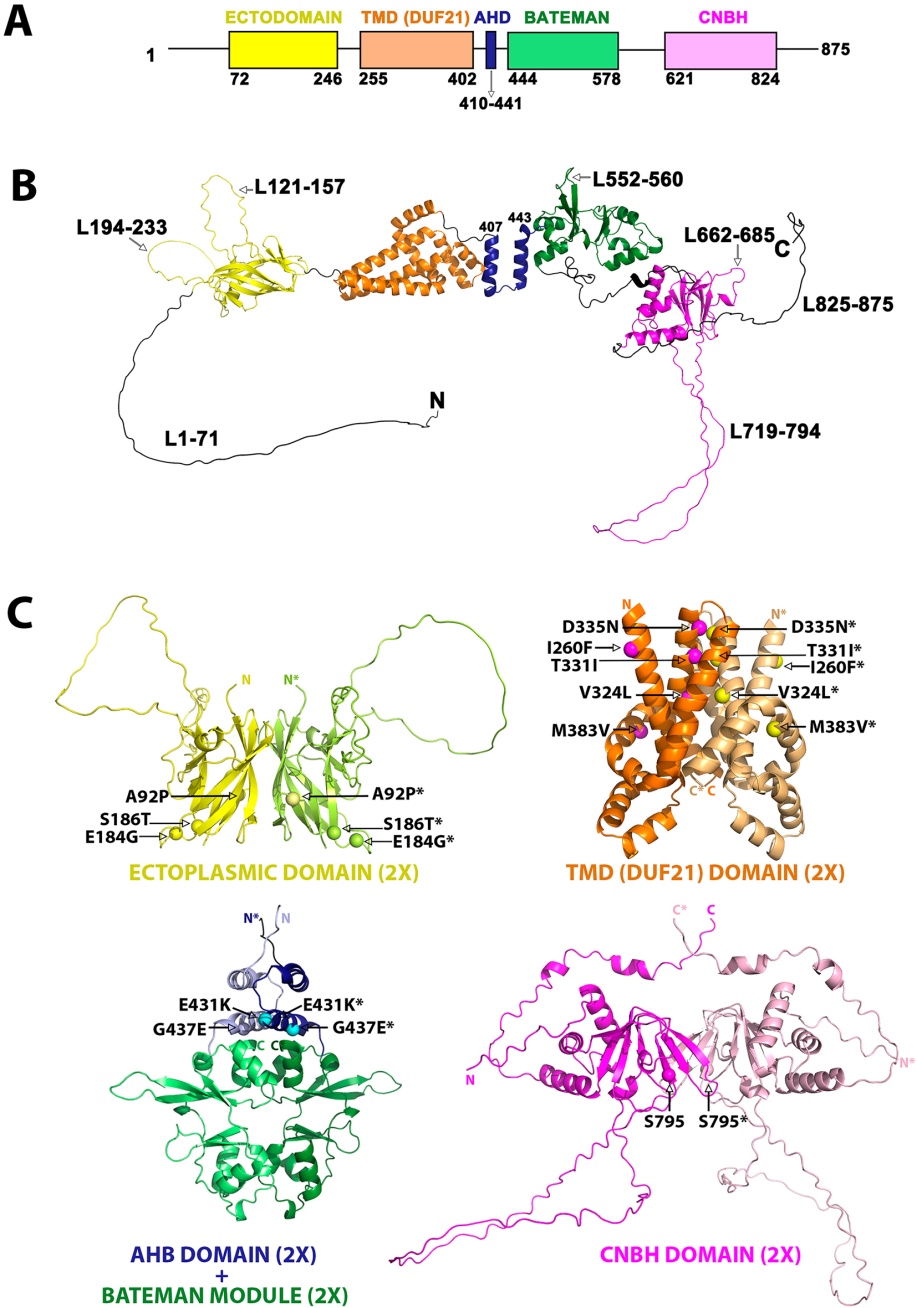
Structure of CNNM2. (**A**) Domain distribution of human CNNM2 (hCNNM2). (**B**) 3D-structure of the hCNNM2 monomer, predicted with AlphaFold-2 and based on the available crystallographic knowledge. (**C**) 3D-structure of the dimeric symmetric assemblies formed by the independent CNNM2 domains resulting from combining AlphaFold-2 predictions (ectodomain and DUF21 domain) with crystallographic data (Bateman module, PDB ID: 4IYS, 4P1G, 4IY0, 4P1O, 4IY4; CNBH PDB ID: 6DJ3; Bateman module & CNBH PDB ID: 6N7E). The interdomain linkers are not represented as the actual relative orientation between the protein domains dimers remains unclear.

To gain further insight into the pathogenic effect of each variant, we analysed their impact on the protein's structure. CNNM2 is composed of four independent domains, the N-terminal extracellular domain (ectodomain), a transmembrane domain of unknown function 21 (DUF21), a Bateman module and the cyclic nucleotide binding homology (CNBH) domain (Fig. 2A). These domains are linked by extensive polypeptide segments of varying lengths that lack secondary structure. In addition, both the ectodomain and the CNBH domain contain extensive unstructured loops (Fig. 2B). All these flexible segments present a significant challenge in crystallising the CNNMs. Despite this, combining the available crystal structures with structural prediction methodologies allows various characteristics of the CNNM2 structure to be solved. For example, CNNM2 forms dimers with partial twofold symmetry between equivalent domains (Fig. 2C), highlighting the importance of modelling the effects of variants on these dimeric entities.

#### p.A92P

**Figure 3:**
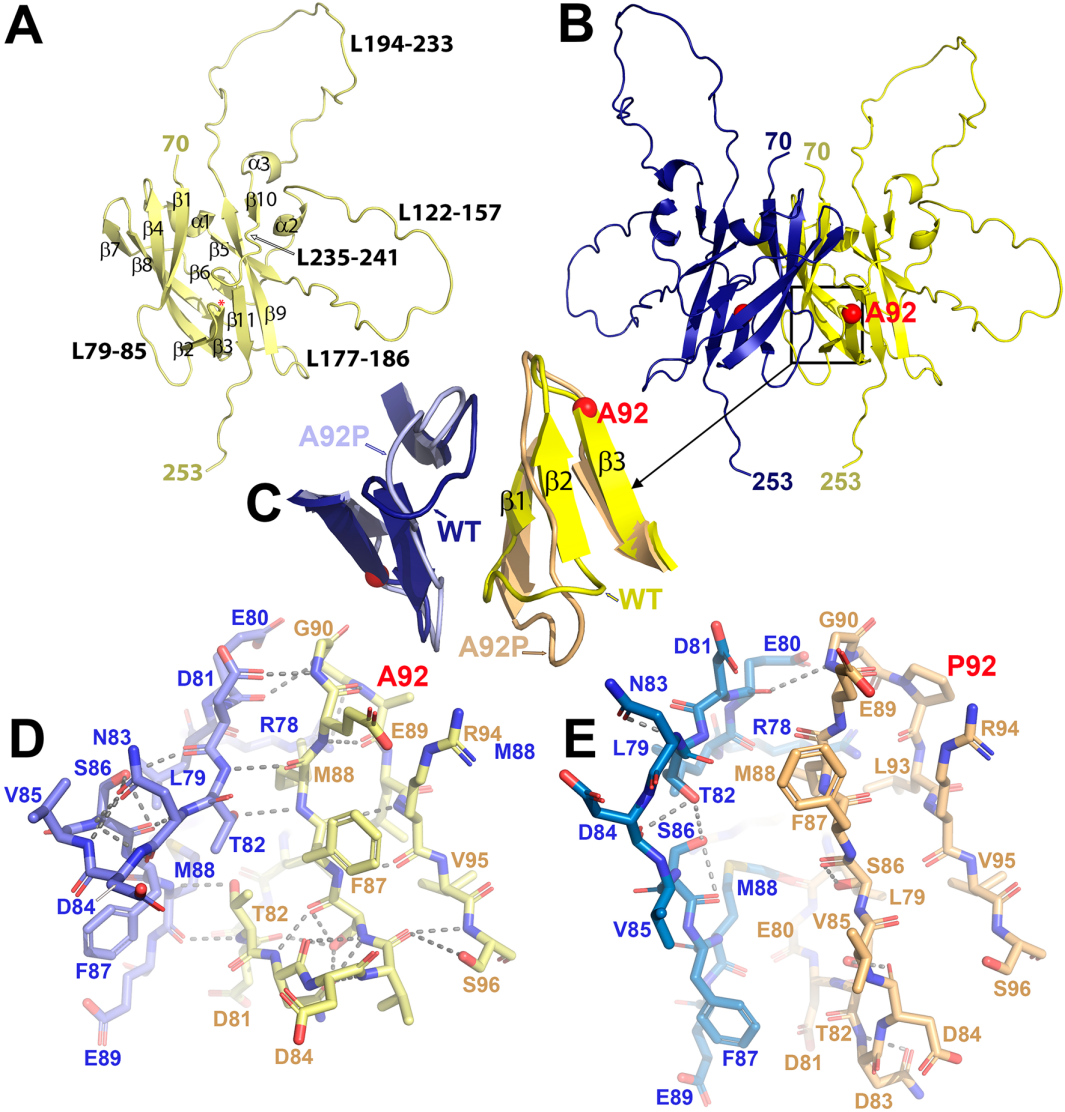
Structure of the CNNM2 ectodomain. (**A**) AF2-predicted 3D-model of CNNM2_Ectodomain_. (**B**) AF2-predicted dimer of the CNNM2_Ectodomain_. (**C**) Zoomed-in view of the residue A92 neighbourhood, illustrating the primary structural changes induced by the substitution of this residue (represented with a red sphere) with a proline. (**D**) Sticks representation of the key amino acid residues and the major contacts (dashed lines) in the vicinity of the residue located at position 92 in the wild-type protein (**D**) and the variant (**E**).

The A92 residue belongs to the ectodomain, which exhibits a β-barrel fold formed by eleven β-strands split into two opposing antiparallel β-sheets (↑β1_(73–82)_-↓β4_(98–108)_-↑β8_(170–177)_-↓β7_(164–167)_ and ↓β2_(87–89)_-↑β3_(92–96)_-↑β11_(243–246)_-↓β9_(187–194)_-↑β5_(118–121)_) (Fig. 3A). The p.A92P substitution induces a conformational change in the polypeptide chain at the start of strand β3 (Fig. 3B). This leads to a slight reorientation and reduction in the length of the strand, which changes the interactions between strand β3 and the adjacent strand β2. Consequently, strand β2 loses its secondary structure and takes on loop-like characteristics, which in turn influences the shortening of strand β1 (Fig. 3C). This sequential chain of local distortions has implications for the interaction between the ectodomains of complementary subunits, particularly involving strand β1. In the p.A92P variant, the β1 strands of complementary subunits exhibit a noticeable separation, compromising their association within the dimer and likely disrupting the normal function of the ectodomain (Fig. 3D,E).

#### p.V324L, p.D335N and p.M383V

Since the DUF21's structural knowledge is confined to a select pair of bacterial homologs within the CNNM family^38,47^, the investigation of variants p.V324L, p.D335N and p.M383V required predicting the three-dimensional structure of this module in hCNNM2 (CNNM2_DUF21_, residues 252–410). Our calculation showed that the CNNM2_DUF21_ dimer closely resembles the overall structure observed in bacteria, with small differences in the orientation/angles of some secondary elements (Fig. S2). Each CNNM2_DUF21_ chain is composed of three antiparallel transmembrane α-helices (TM1-3) that run perpendicular to the cell membrane. Additionally, there are two shorter intracellular α-helices (α1, α2) that are perpendicular to the TMs. Surrounding the outer region of the TM helices, there is a belt-like juxtaposed membrane α-helix (JM), perpendicular to the TMs. In terms of the primary structure, these elements follow the sequence TM1-α1-α2-TM2-TM3-JM (Fig. S2).

**Figure 4:**
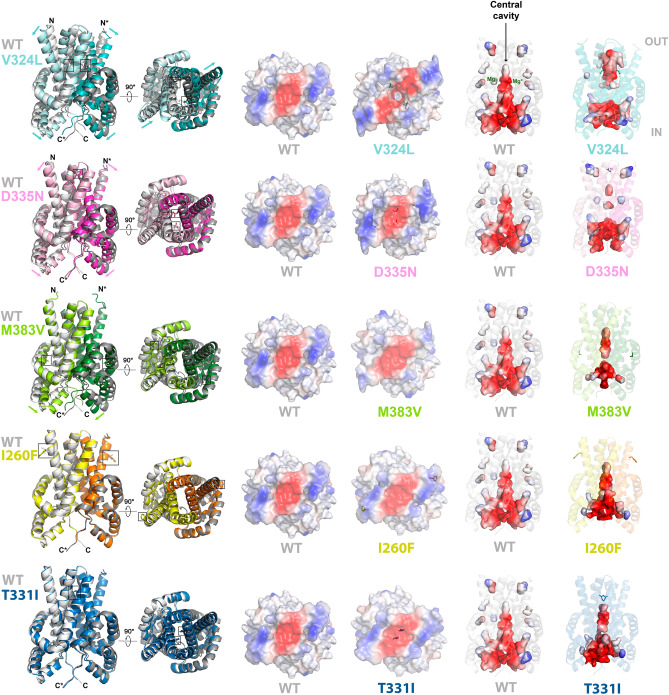
Structural effect of variants V324L, D335N, M383V, I260F and T331I on CNNM2_DUF21_. Residues affected by the variants are in sticks. IN and OUT indicate the intracellular and extracellular sides of the DUF21 domain, respectively.

At the monomeric level, only subtle effects are observed in the p.V324L, p.D335N and p.M383V variants. The most significant deviations are found in the TM helices of the p.V324L species, as reflected by the root mean square deviation (RMSD) value resulting from aligning its three-dimensional structure with that of the native enzyme (Fig. S3). At the dimeric level, much more significant changes are observed. The DUF21 region self-associates forming a membranous bundle perpendicular to the membrane related by twofold symmetry (Figs. S3 and S4). Based on findings in the bacterial homologue TpCorC, the CNNM2_DUF21_ dimer adopts an *inward-opening* conformation, where the cytoplasmic side is exposed to the solvent, while the extracellular side is closed off^38^. Under this *inward-opening* conformation, the complementary subunits create a large electronegative cavity in the center of the dimer, that presumably facilitates the access of Mg^2+^ ions (Fig. S4). The p.V324L, p.D335N and p.M383V variants induce a change in the relative angle of the TM and α1-α2 helices, which dramatically affects the volume of the central electronegative cavity (Fig. 4). In the variants, the cavity is interrupted approximately halfway through its course, disconnecting its cytoplasmic part from the extracellular zone, thus presumably interrupting the Mg^2+^ transport through the membrane. This resembles an inversion of the *inward-opening* to the *outward-opening* conformation, where the TM helices move away from the center of the cavity, while the α1-α2 helices approach it (Fig. 4). These helical shifts are most pronounced in variant p.V324L, where the cavity is divided into two separate halves, disconnected from each other at the location of the variant. Though the helical shifts may appear subtler for p.D335N and p.M383V, the portion of the cavity facing the extracellular space is completely closed, leaving only the cavity in contact with the cytoplasm (Movies SM1–3).

In summary, it appears that variant p.D335N, located closer to the extracellular end of the TM domains, irreversibly stabilises the *inward-opening* conformation. On the other hand, the p.M383V variant primarily impacts the orientation of the JM, α1 and α2 helices, while seemingly not causing significant alterations in the TM helices. Consequently, the interior electronegative cavity of the dimer experiences the least amount of impact compared to the other two variants. Finally, the p.V324L variant, situated midway along the TM helices, tends to promote the *outward-opening* conformation. However, this variant places the inward-to-outward transformation mechanism, which presumably facilitates the passage of Mg^2+^ ions, in an intermediate, non-functional state.

#### p.E431K and p.G437E

Residues E431 and G437 are part of the acidic helical bundle (AHB) domain, which consists of two antiparallel helices, αA_(411–421)_ and αB_(429–443)_, and serves as a link between DUF21 and the cytoplasmic Bateman module (Fig. S5A). When the protein dimerises, these helices align in an antiparallel manner with their counterparts from the complementary subunit, forming a compact and symmetric helical cluster.

The p.E431K substitution inverts the electrostatic features of the environment of this residue and introduces an electrostatic repulsion with electropositive amino acids located at the ends of the αB helices of the AHB helical cluster, like R411 and R442 (Fig. S5B–D). The most notable consequence is the shift in the orientation of the complementary αB helices, which affects the orientation of the Bateman module with respect to the AHB domain in both monomers (Fig. S5E).

Like the E431 residue, G437 is located within the αB helix of the AHB domain. However, in this case, it is oriented towards the last α-helix (residues 569–577) of the CBS2 motif of the Bateman module (Fig. S6). Structurally, the effect of p.G437E is less dramatic than the p.E431K variant, but it still exerts a distortion in the communication between the two aforementioned domains. The presence of the glutamate facilitates the formation of a previously nonexistent electrostatic interaction between this residue and the lysine at position 578 (Fig. S6A). This new interaction triggers a cascade of slight displacements of the surrounding amino acid side chains that finally results in a displacement of the whole Bateman module with respect to the AHB domain (Fig. S6B). Since the AHB domain is responsible for transmitting changes from the Bateman module to the transmembrane region, it is reasonable to anticipate that these structural alterations impact the transport mechanism of Mg^2+^ ions across the membrane.

#### p.S795*

The CNBH domain consists of two distinct regions. One comprises three α-helices and connects the CNBH domain to the Bateman module and the second contains two β-sheets composed of four (↑β1_(659–662)_-↓β7_(802–808)-_↑β2_(685–691)_-↓β5_(710–713)_) and three (↑β6_(794–798)_-↓β3_(694–698)_-↑β4_(703–707)_) strands (Fig. S7A). S795 is located at the second β-sheet, at the beginning of strand β6, right after the long loop comprising residues 720–794 (Fig. S7A). The strand β6 is inserted between strand β3 and loop 662–684, which in turn interacts through residues L676 and Y677 within a hydrophobic pocket participated by residues from the first β-sheet (L662, V684, Y685, I689, Y713, L802 and F804). It is evident that the deletion of the polypeptide chain starting from residue 795 completely destabilises the integrity of this structural arrangement, making it unfunctional and much more susceptible to aggregation and/or degradation.

#### p.E184G, p.S186T, p.I260F, p.T331I

Lastly, we investigated the variants p.E184G, p.S186T, p.I260F, and p.T331F, which did not exhibit a loss-of-function effect in the transport assay. Residues E184, S186 and I260 are located in regions distanced from the core of their respective domains, projecting their side chains outward from the protein structure (Figs. 4 and S8). This explains the minimal disruption caused by their substitution, preserving the overall folding integrity of the domain and the protein's dimerisation. Conversely, residue T331, situated within the C-terminal region of TM2, places its side chain inward within the DUF21 domain dimer, directly opposing its equivalent residue in the complementary subunit (Fig. 4). The p.T331I substitution enhances the hydrophobic environment within the C-terminal region of the TM2 helix (Fig. S8D). Within the dimer, this newly acquired characteristic strengthens the connections between the TM2 helices of complementary subunits, augmenting the contributions of residues I332, I352, V324, and A344 in the native protein. Unlike mutations p.V324L, p.D335N, or p.M383V, the direct effect of the p.T331I substitution does not disrupt the dimer's central cavity, but stabilises one of the two essential conformations needed to facilitate ion transport. More precisely, the p.T331I substitution promotes the *inward* rather than the *outward* conformation of the DUF21 module dimer (Fig. 4). This restricted flexibility is likely to impede the transit of metal cations across the cell membrane.

## Discussion

In this study, we have identified five new heterozygous *CNNM2* variants (p.A92P, p.V324L, p.M383V, p.E431K, p.G437E) and provided further evidence for the pathogenicity of two reported variants (p.D335N, p.S795*), resulting in a likely pathogenic or pathogenic classification of all seven variants according to the ACMG guidelines (Table S2). Firstly, this is based on the presence of phenotypes that overlap with previous reports, most consistently hypomagnesaemia or low-normal serum Mg^2+^. Secondly, a ^25^Mg^2+^ uptake assay shows a significant reduction in CNNM2-mediated Mg^2+^ uptake for all seven variants. Lastly, all variants are predicted to affect CNNM2 function by structural modelling. Altogether, these findings will help improving the classification of *CNNM2* variants and provide further insight into the genotype–phenotype relationship of *CNNM2*-related disorders.

The phenotype of individuals with pathogenic *CNNM2* variants can be highly variable. In previous reports of multiple affected individuals, the predominant phenotype was hypomagnesaemia, seizures, mild to moderate ID (severe in homozygous cases) and obesity^2,5^. In the majority of the cases described here, the extrarenal symptoms are mostly absent or mild. Differences in phenotype can be partially explained by the results of the functional assay. For example, the three affected individuals with variants that show a relatively high residual functionality in the ^25^Mg^2+^ assay (cases 1, 3 and 7; p.A92P, p.M383V and p.D335N) present with the highest Mg^2+^ levels (0.60–0.73 mmol/L) compared to the other cases. However, variants with similar functional effects do not always cause similar phenotypes. A striking example of this is the absence of obvious extrarenal phenotypes in case 6 (p.S795*) compared to an individual with a comparable nonsense variant (p.R797*), who presented with seizures, ID, obesity, autism and minor brain malformations^4,5^. These findings indicate that additional (genetic) factors have to be present for such phenotypes to occur.

**Figure 5:**
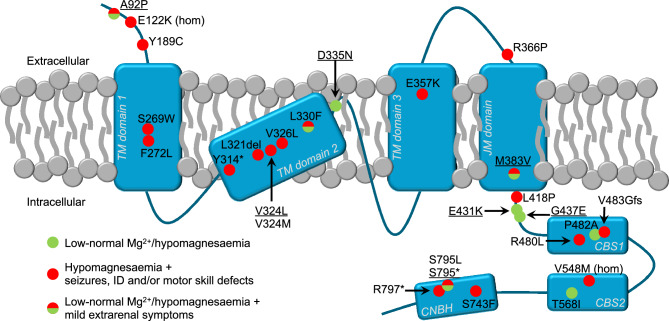
Association between variant location within the CNNM2 protein and disease severity. Variants from this study are underlined. CBS, cysthatione-β-synthase domains; CNBH, cyclic nucleotide binding homology; hom, homozygous; ID, intellectual disability; JM, juxtaposed membrane α-helix; TM, transmembrane.

The protein domain in which the variant occurs also plays a role in genotype–phenotype correlations. Variant p.A92P is located in the ectodomain and seems to only partially affect CNNM2 function. The p.V324L and p.M383V variants are in transmembrane regions of the highly conserved DUF21, which has previously been postulated to be associated with the most severe phenotype^8^. Indeed, p.V324L strongly affects CNNM2-mediated Mg^2+^ transport and causes a severe phenotype of hypomagnesaemia, seizures, obesity and ID, which is in line with a previous case wherein the same residue was affected (p.V324M)^5^. p.M383V, however, causes a less severe phenotype, with hypomagnesaemia and mild ID in the index case and hypomagnesaemia in the mother. This variant also shows more residual functionality in the ^25^Mg^2+^ assay, indicating it is not as deleterious as other DUF21 variants. Indeed, the structural modelling shows that p.M383V impacts the transmembrane structure of both the monomer and the dimer less severely than p.V324L. The p.E431K and p.G437E variants are in the intracellular AHB domain and both severely affect Mg^2+^ transport capacity. Despite this, they cause a less severe phenotype without ID, seizures or obesity. These data suggest that only variants in the transmembrane DUF21 that severely impact CNNM2 function (< 20% functionality) consistently cause all features of *CNNM2*-related disorder, while variants located in the extra- and intracellular regions and/or variants with more residual functionality are not always associated with extrarenal symptoms (Fig. 5).

In addition to the new variants, we also included variants previously described by Garcia-Castano et al*.* for functional analysis. As expected, the truncating variant p.S795* disrupts CNNM2-mediated Mg^2+^ uptake, confirming it is causative for the hypomagnesaemia in case 6. Interestingly, despite the absence of the most common *CNNM2*-related symptoms in case 7, the p.D335N variant also showed a reduction in Mg^2+^ uptake. This variant may therefore contribute to the observed low-normal Mg^2+^ levels. Although this variant is within the DUF21 domain, it shows a relatively large residual uptake capacity similar to p.M383V, which could explain the mild phenotype. This is in line with the structural data, which predicts that p.D335N has a less severe effect on the TM helices compared to p.V324L. These findings show that functional analysis and elaborate in silico predictions improves the classification of previously reported variants.

In case 4 and in both cases described by Garcia-Castano et al*.*, hyperparathyroidism and disturbed Ca^2+^ homeostasis was observed. It should be noted that both cases 4 and 7 have additional variants in genes associated with PTH and Ca^2+^ regulation (*SLC9A3R1* and *VDR*) that may explain these phenotypes. Indeed, the daughter of case 4 does not have the *SLC9A3R1* variant and has normal PTH levels, while family members of case 7 have hypocalciuria and the variant in *VDR*, but not in *CNNM2*. However, no variants in known PTH/Ca^2+^ genes were found in case 6 and previous reports of individuals with *CNNM2* variants have also observed hyperparathyroidism^3^, hypocalciuria^1,3^ and/or hypocalcaemia^5,7,10^, while increased serum Ca^2+^ is observed in *Cnnm2*^+/−^ mice^16^. In individuals with variants in *TRPM6* and *TRPM7*, encoding Mg^2+^ channels crucial for uptake of Mg^2+^ in the DCT and other tissues, it has been shown that hypocalcaemia can occur secondary to hypomagnesaemia^48–50^. However, these individuals typically have lower serum Mg^2+^ levels than observed in individuals with *CNNM2* variants and the prevalence of hypocalcaemia is too rare to conclude it is directly related. Moreover, two cases described here and the *Cnnm2*^+/−^ mice have hypercalcaemia rather than hypocalcaemia, further complicating the interpretation of this association. Although it is unclear whether the sporadic observations of hyperparathyroidism and Ca^2+^ disturbances are directly linked to CNNM2 function, these findings indicate that *CNNM2* variants should be considered earlier in individuals with unsolved Ca^2+^ and PTH phenotypes.

Functional testing is important to determine the pathogenicity of newly identified variants. In line with previous studies, we used a ^25^Mg^2+^ uptake assay to provide functional evidence for the pathogenicity of *CNNM2* variants^2,5^. As shown here, the level of residual Mg^2+^ uptake capacity correlates well with the severity of the hypomagnesaemia. We advise the use of functional testing for future variants that are identified in individuals with hypomagnesaemia. It should be noted, however, that this assay mainly focuses on the role of CNNM2 in Mg^2+^ reabsorption. ID, motor skill defects and obesity have been observed in individuals with *CNNM2* variants but without hypomagnesaemia^7^, indicating that these extrarenal symptoms occur (partially) independently of the hypomagnesaemia. The role of CNNM2 in brain development and metabolism has not been extensively studied, but polymorphisms in *CNNM2* have regularly been associated with schizophrenia^51–53^, obesity^54^ and cardiovascular disease^54–57^, indeed indicating additional functions. We cannot exclude that some variants (such as p.E184G, p.S186T, p.I260F and p.T331I) may contribute to neuronal defects, while maintaining Mg^2+^ transport function. This consideration is especially important for p.T331I, which, in contrast to p.E184G, p.S186T and p.I260F, seems to cause significant alterations in the 3D structure and is predicted to be pathogenic by 3 out of 5 prediction tools. However, functional assays specifically looking at brain development are currently not available for *CNNM2* variants.

In conclusion, we have identified new pathogenic variants in *CNNM2*, which contributes to an enhanced understanding of the phenotypes of *CNNM2*-related disorders. The most striking finding is the absence of seizures, ID, and obesity in the majority of the cases described here, suggesting that variants may independently affect renal and extrarenal functions of CNNM2. Future studies may further unravel the exact role of CNNM2 in Mg^2+^ reabsorption as well as brain development, metabolism and potentially PTH/Ca^2+^ homeostasis.

### Supplementary Information

Supplementary Information.

Supplementary Video S1.

Supplementary Video S1.

Supplementary Video S2.

Supplementary Video S2.

Supplementary Video S3.

Supplementary Video S3.

## Data Availability

The genetic variants identified in this study were deposited in ClinVar (accession numbers SCV004032141-SCV004032149; p.T331I and p.M383V previously reported under accession numbers SCV002581907 and SCV001502653 respectively). All relevant data are within the manuscript and its supplementary files. Additional data that support the findings of this study are available from the corresponding author upon reasonable request.
