# Supplementary material for: Hypomagnesaemia with varying degrees of extrarenal symptoms as a consequence of heterozygous CNNM2 variants
Source: Sci Rep. 2024 Mar 22;14:6917. doi: 10.1038/s41598-024-57061-7 (PMC10959950; doi:10.1038/s41598-024-57061-7)
Supplement: Supplementary file 1 — Supplementary Information. [file 41598_2024_57061_MOESM1_ESM.pdf]

SUPPLEMENTARY INFORMATION

Hypomagnesaemia with varying degrees of extrarenal symptoms as a consequence of heterozygous *CNNM2* variants

Willem Bosman, Gijs A. C. Franken, Javier de las Heras, Leire Madariaga, Tahsin Stefan Barakat, Rianne Oostenbrink, Marjon van Slegtenhorst, Ana Perdomo-Ramírez, Félix Claverie-Martín, Albertien M. van Eerde, Rosa Vargas-Poussou, Laurence Derain Dubourg, Irene González-Recio, Luis Alfonso Martínez-Cruz, Jeroen H. F. de Baaij, Joost G. J. Hoenderop

Table S1. Variant characterisation.

mRNA	Protein	GnomAD	Exome Variant Server	GME Variome	PhyloP (cut-off ≥7.367)	CADD PHRED (cut-off ≥25.3)	PolyPhen (cut-off ≥0.978)	VEST4 (cut-off ≥0.764)	Mutation-Taster	Ref
274G>C	A92P	Not found	Not found	Not found	4.729	19.28	0.734	0.193	Benign	
970G>C	V324L	Not found	Not found	Not found	9.807	26.6	0.954	0.868	Deleterious	
1003G>A	D335N	Not found	Not found	Not found	9.807	23.6	0.229	0.815	Deleterious	[4]
1147A>G	M383V	Not found	Not found	Not found	9.324	23.4	0.423	0.887	Deleterious	
1291G>A	E431K	Not found	Not found	Not found	9.852	29.6	0.675	0.959	Deleterious	
1310G>A	G437E	Not found	Not found	Not found	9.852	28	0.897	0.941	Deleterious	
2384C>A	S795*	Not found	Not found	Not found	5.995	44	-	0.877	Deleterious	[4]
551A>G	E184G	Not found	Not found	Not found	5.017	25.1	0.985	0.445	Deleterious	
557G>C	S186T	Not found	Not found	Not found	6.312	19.7	0.801	0.384	Deleterious	[4]
778A>T	I260F	Not found	Not found	Not found	7.101	24	0.408	0.814	Deleterious	[4]
992C>T	T331I	Not found	Not found	Not found	7.771	24.1	0.100	0.926	Deleterious	

Values above the cut-off value of pathogenicity are underlined

Table S2. ACMG classification

mRNA	Protein	Evidence of pathogenicity				Classification
		Supporting	Moderate	Strong	Very strong	
274G>C	A92P	PP2	PM2 + PM6[a]	PS3	-	Likely pathogenic
970G>C	V324L	PP2 + PP3 + PP4	PM2 + PM5	PS2 + PS3	-	Pathogenic
1003G>A	D335N	PP2	PM2 + PM6[a]	PS3	-	Likely pathogenic
1147A>G	M383V	PP2 + PP3	PM2	PS3	-	Likely pathogenic
1291G>A	E431K	PP2 + PP3	PM2	PS3	-	Likely pathogenic
1310G>A	G437E	PP2 + PP3	PM2	PS2 + PS3	-	Pathogenic
2384C>A	S795*	PP1 + PP3	PM2	PS3 + PVS1_strong[b]	-	Pathogenic

[a]Confirmed *de novo*, but aspecific phenotype
[b]Nonsense variant in a critical domain, but not predicted to undergo nonsense-mediated decay

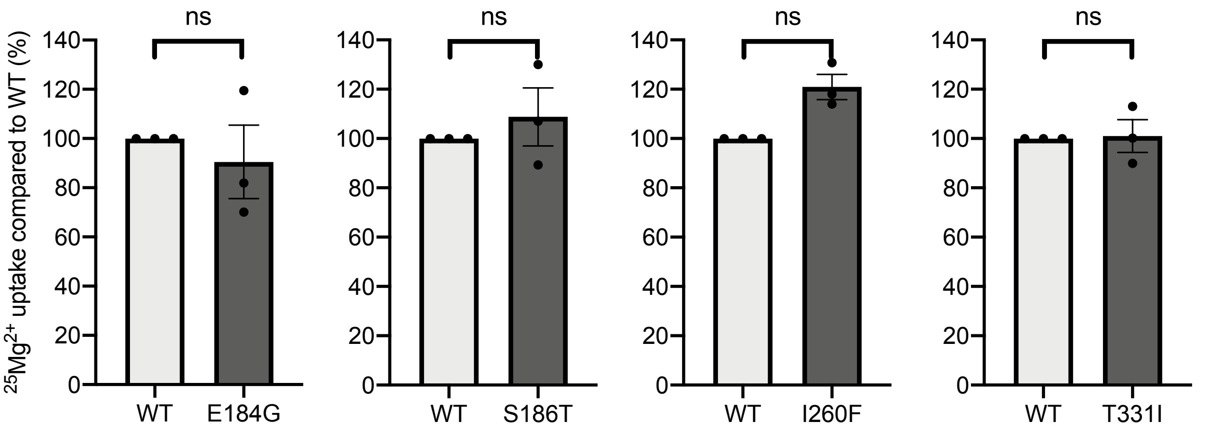

Figure S1. Unaffected Mg^{2+} uptake of four variants compared to WT CNNM2. Percentage of $^{25}Mg^{2+}$ uptake capacity of each variant compared to WT. Data were obtained from 3 independent experiments and presented as mean ± SEM. ns=not significant.

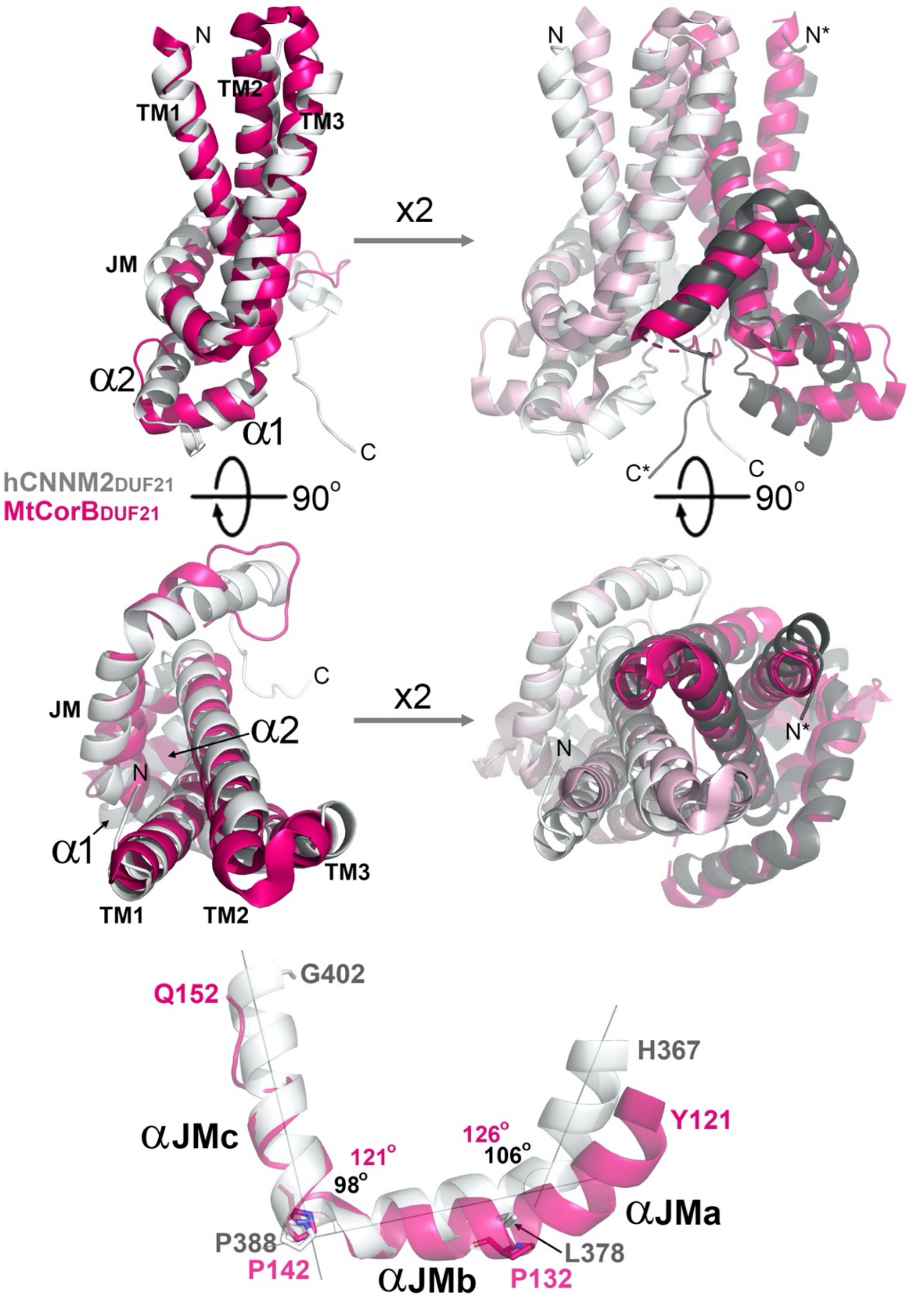

Figure S2. Structure of CNNM2$_{DUF21}$ and MtCorB$_{DUF21}$. 3D-Alignment of the AF2-predicted model of CNNM2$_{DUF21}$ (grey) and MtCorB$_{\mathbf{DUF21}}$ (pink) (PDB ID 7M1T) in their monomeric (left) and dimeric (right) forms. (bottom) 3D-Alignment of the JM helices of CNNM2$_{DUF21}$ and MtCorB$_{DUF21}$. The approximate angles between the corresponding JMa, JMb and JMc segments are indicated.

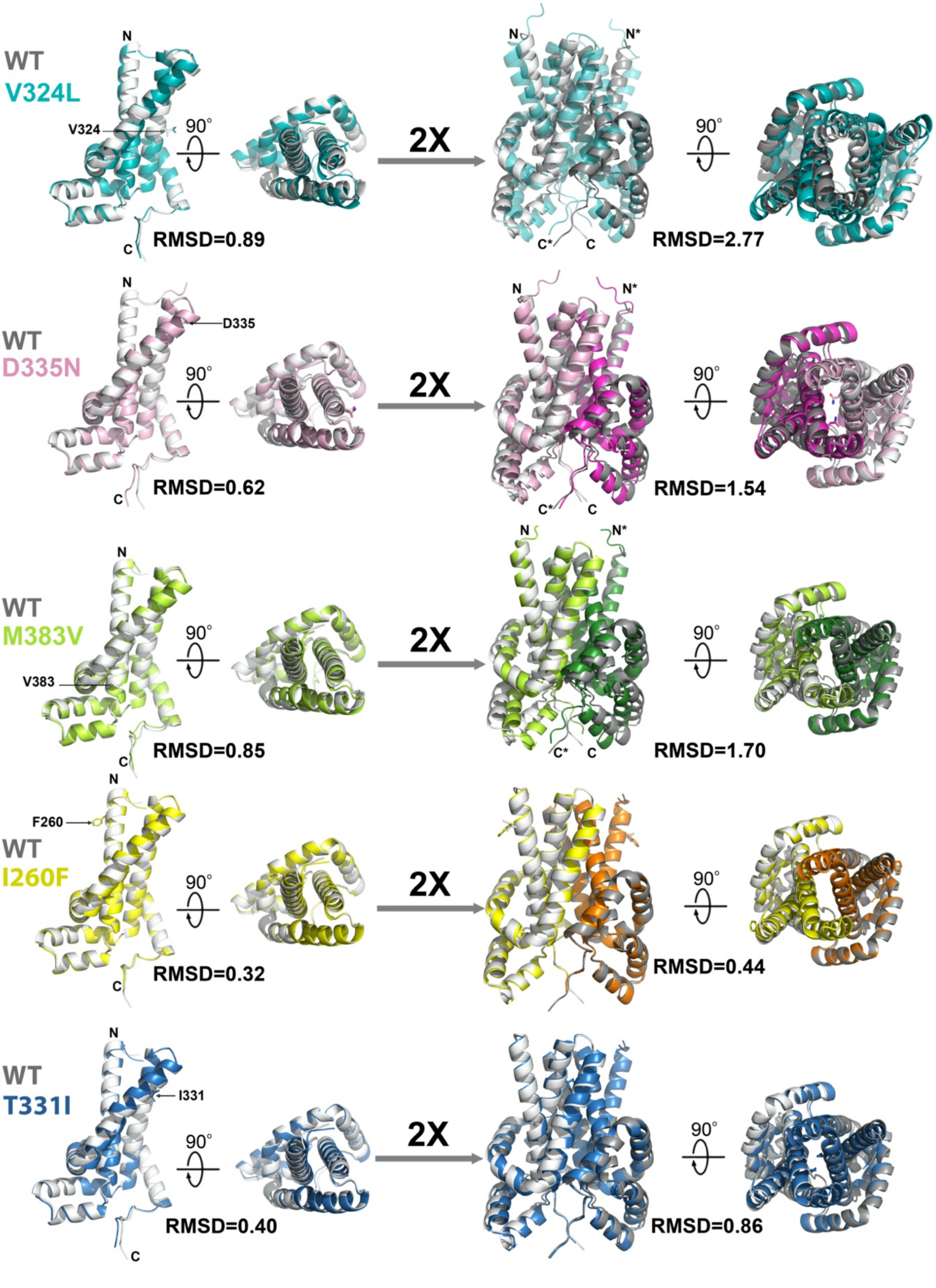

Figure S3. Structural Alignment of the AlphaFold-2 predicted models of CNNM2$_{DUF21}$ vs the pathogenic variants V324L, D335N, M383V, I260F and T331I in their monomeric and dimeric species. RMSD: Root mean square deviation.

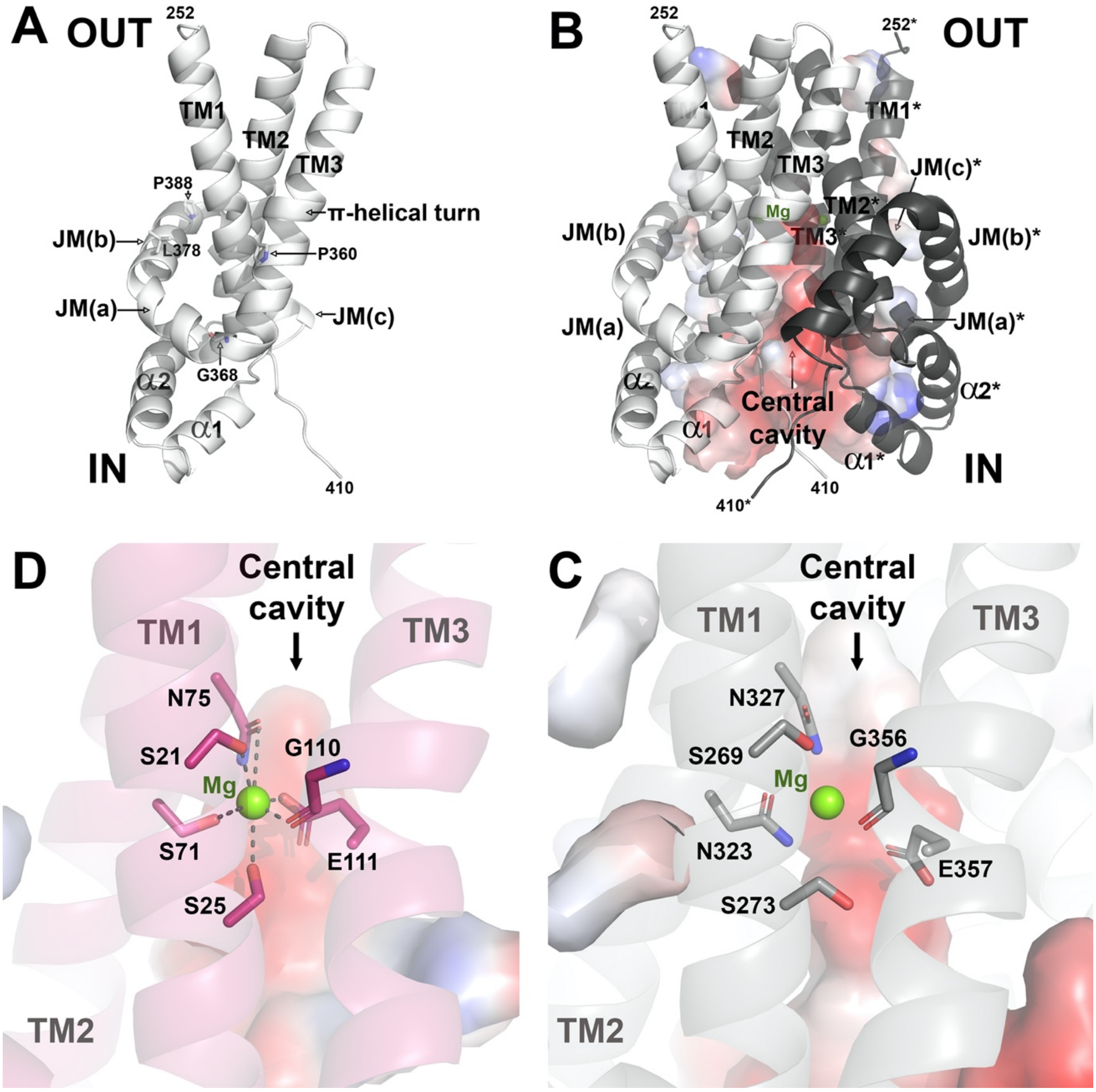

Figure S4. Structure of CNNM2$_{DUF21}$. (**A**) AF2 predicted structure of human CNNM2$_{DUF21}$. (**B**) Dimeric association of CNNM2$_{DUF21}$. The electronegative/electropositive regions are colored in red/blue, respectively. Green spheres indicate the location of Mg^{2+} ions extrapolated from the CNNM2$_{DUF21}$ bacterial homologs. (**C, D**) Zoom-in view of the Mg^{2+} binding site in CNNM2$_{DUF21}$ in humans (**C**) and bacteria (**D**). IN and OUT indicate the intracellular and extracellular sides of the DUF21 domain, respectively.

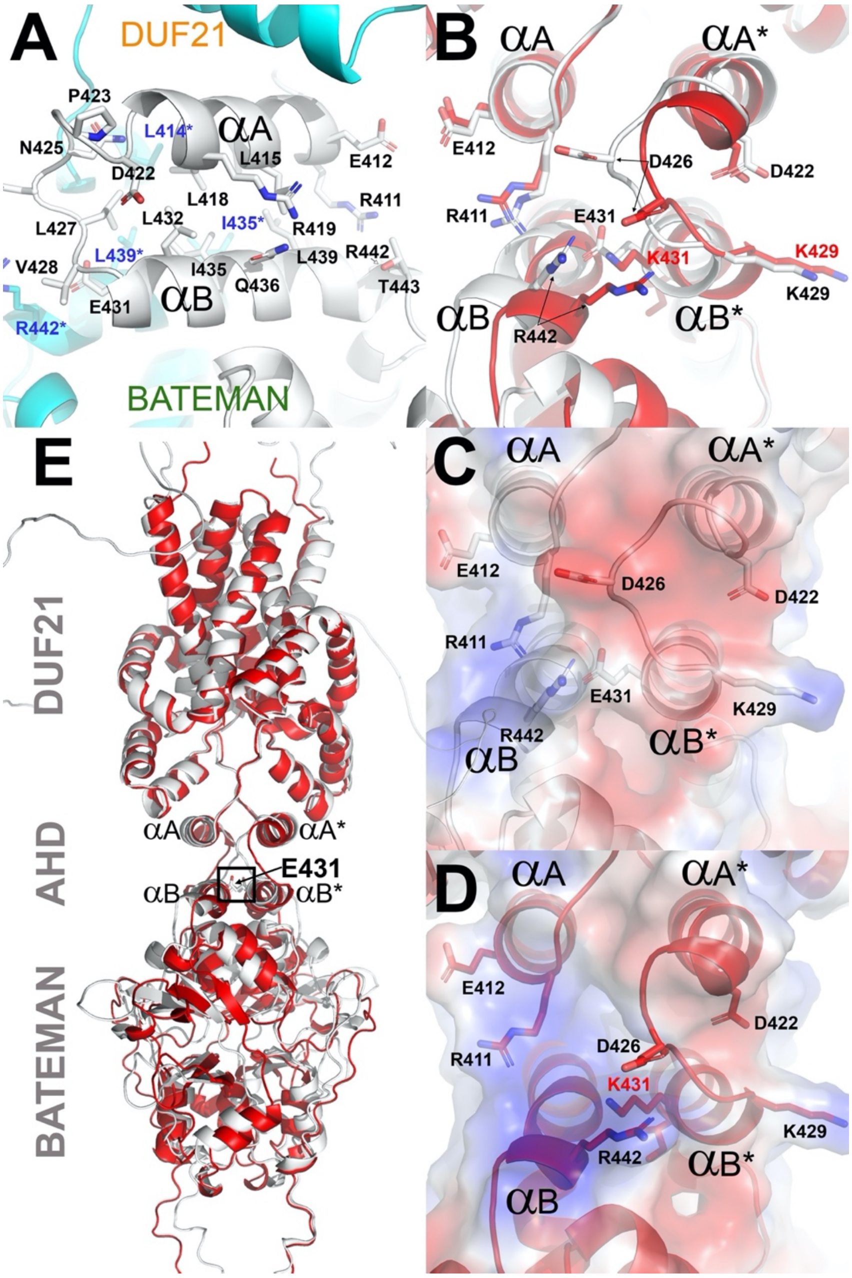

Figure S5. Structural effect of the E431K mutation. (**A**) Main residues involved in the interaction between helices αA and αB within the AHB domain. (**B**) Superimposition of native CNNM2 (grey) and its E431K variant (red) in the vicinity of residue E431 showing the displacement of helix αB. (**C, D**) Electrostatic distribution (positive/negative charges colored in blue and red, respectively) in native CNNM2 and E431K variant. (**E**) Structural superimposition of native CNNM2 (grey) and E431K variant (red), aligned on the DUF21 domain of the dimer (CNBH domain is not represented), showing the displacement of the Bateman module caused by the mutation in the AHB domain. The location of residue E431 is highlighted.

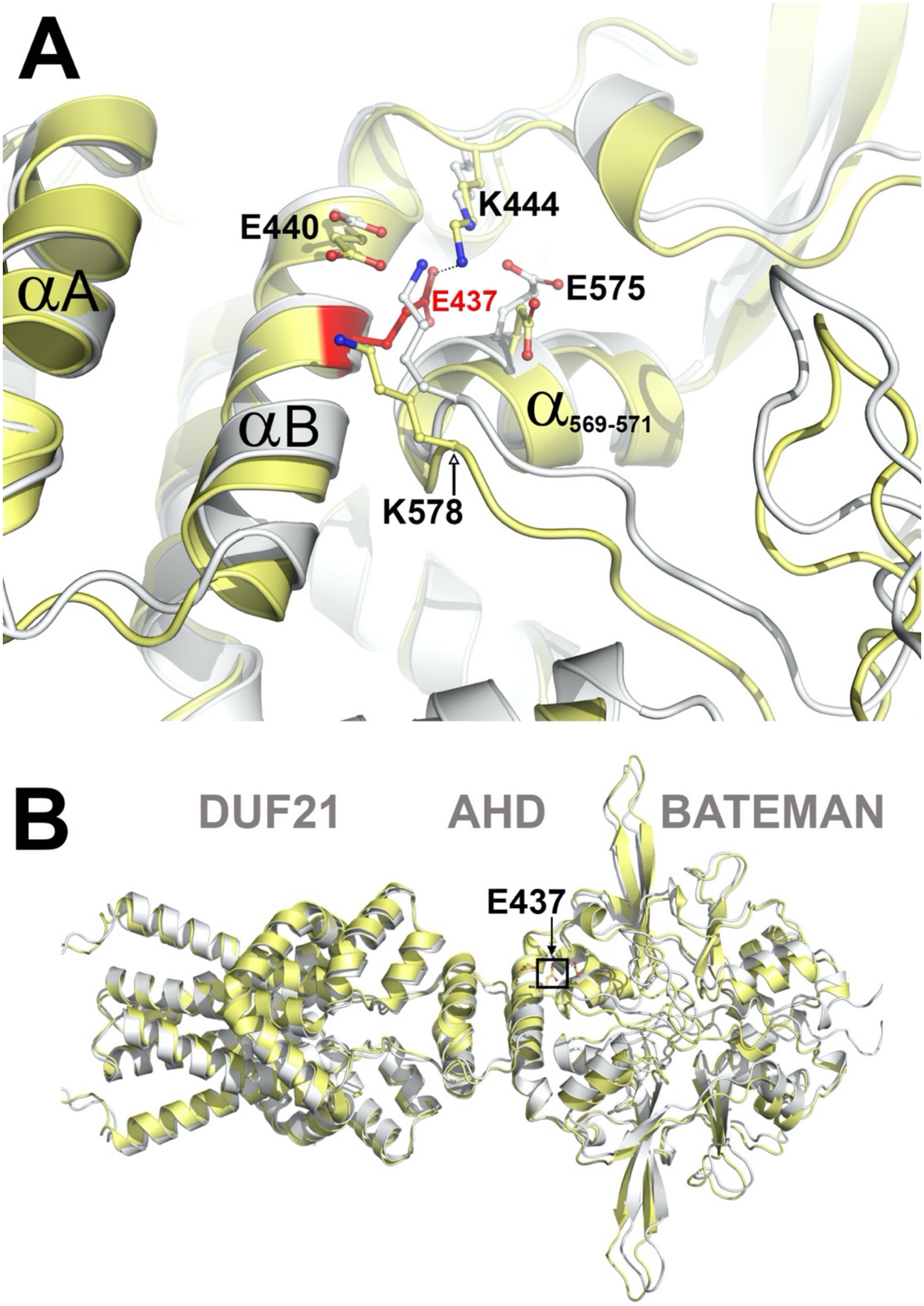

Figure S6. Structural effect of the G437E mutation. (**A**) Main residues involved in the interaction between helix αB and the Bateman module in the native (grey) and in the G437E variant (yellow). The E437 residue is highlighted in red. (**B**) Structural superimposition of native CNNM2 (grey) and G437E variant (yellow), aligned on the DUF21 domain of the dimer (CNBH domain is not represented), showing the displacement of the Bateman module caused by the mutation in helix αB. The location of residue E437 is highlighted.

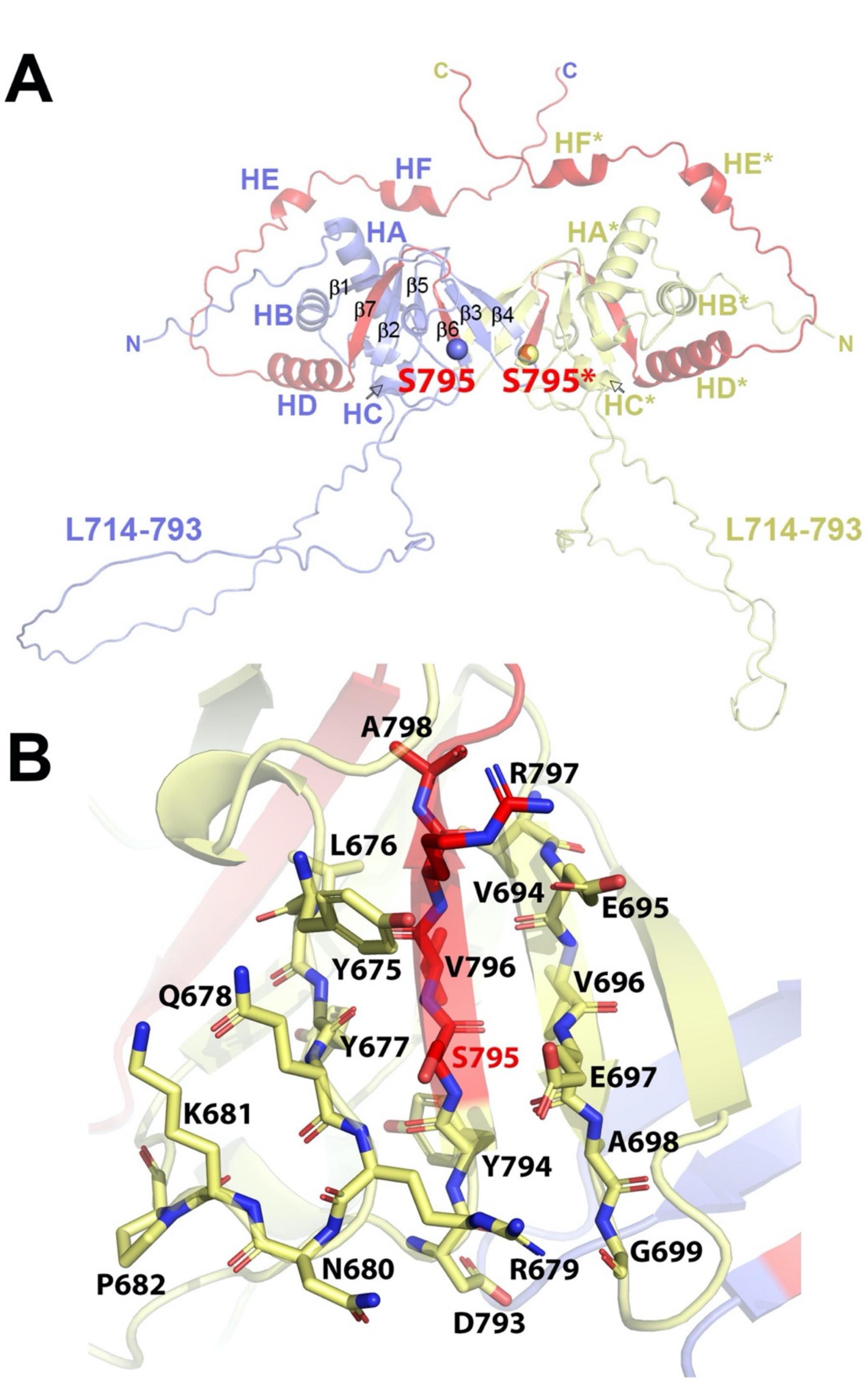

Figure S7. Structure of CNNM2$_{CNBH}$. (**A**) Dimeric association found in the crystal structure of CNNM2$_{CNBH}$ (PDB ID 6DJ3), complemented with AF2 predictions for the internal loop containing residues 662-713 (not visible in the crystals) and the C-terminal disordered tail following the CNBH domain. The polypeptide segment that is absent in the S795* variant is highlighted in red. (**B**) A zoomed-in view of the residue S795 neighborhood. The structural elements absent in the S795* variant are highlighted in red.

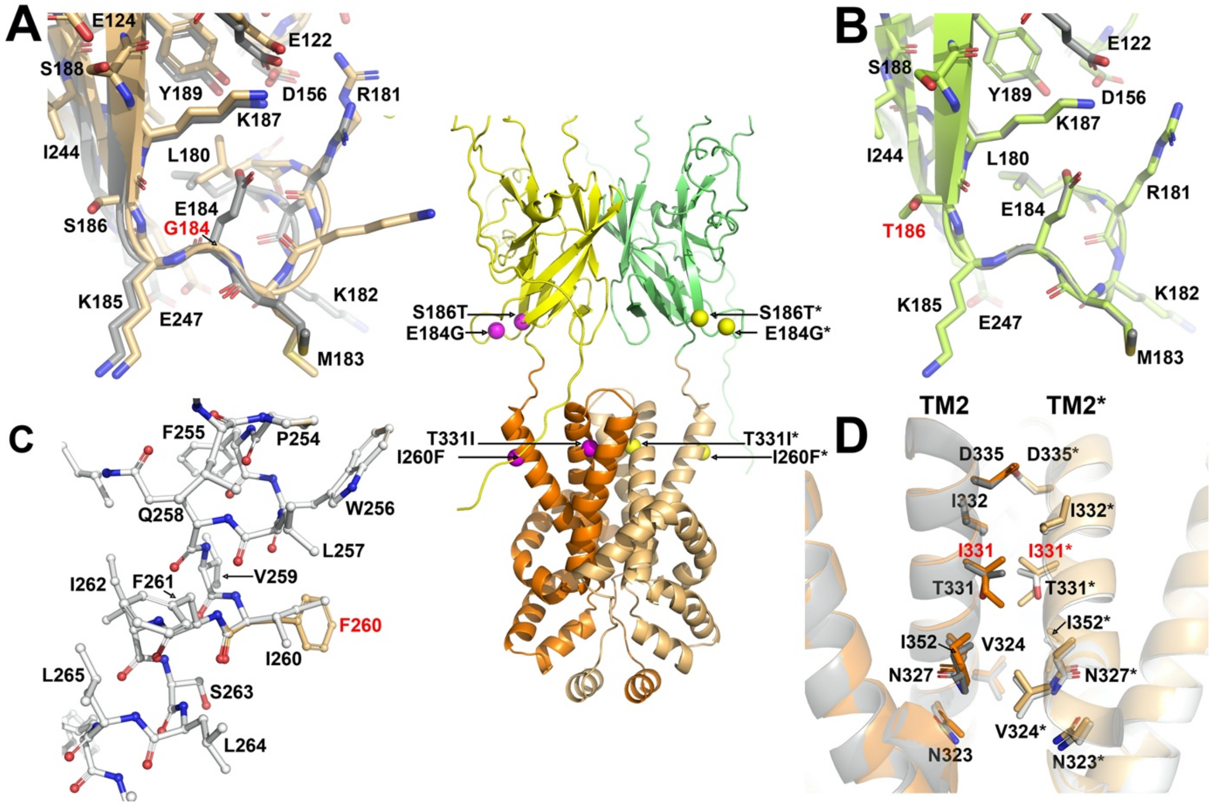

Supplementary Figure S8. Structural effect of variants E184G, S186T, I260F and T331I. (**A** and **B**) Overlap of the ectodomain of the hCNNM2 native protein (grey) with the variants E184G (light orange) and S186T (green) around the relevant residues. (**C** and **D**) Superimposition of the native protein (grey) with its variants I260F and T331I (both in light orange).

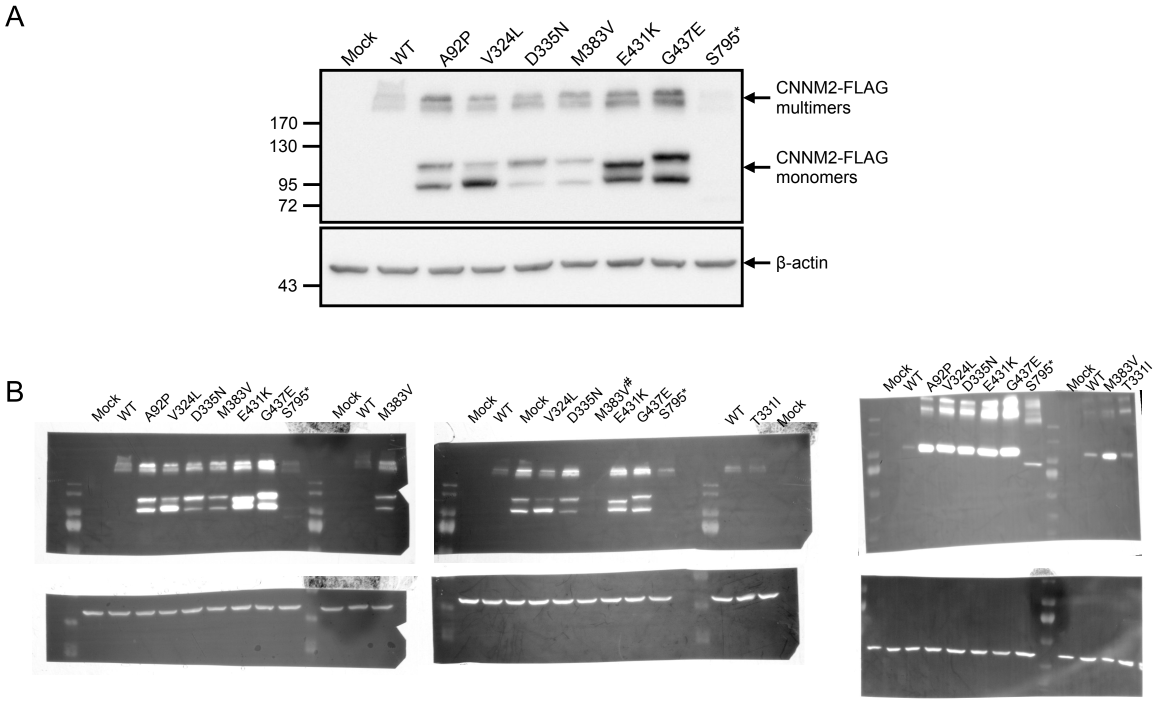

Figure S9. Supporting images for Western blot. (**A**) Lower exposure of the Western blot image. (**B**) Uncropped images of the CNNM2-FLAG and β-actin blots.
